# Identification of immunogenic cell death-related damage-related molecular patterns (DAMPs) to predict outcomes in patients with head and neck squamous cell carcinoma

**DOI:** 10.1007/s00432-024-05779-2

**Published:** 2024-05-07

**Authors:** Jiayi Zhang, Xinzhan Shi, Mengqi Wang, Rundong Zhai, Mengyao Wang, Zizhen Gong, Zihui Ni, Teng Xu, Weiwen Zhu, Laikui Liu

**Affiliations:** 1 Department of Basic Science of Stomatology, The Affiliated Stomatological Hospital of Nanjing Medical University, Jiangsu, China; 2 Department of Oral and Maxillofacial Surgery, The Affiliated Stomatological Hospital of Nanjing Medical University, Jiangsu, China; 3 State Key Laboratory Cultivation Base of Research, Prevention and Treatment for Oral Diseases, Nanjing Medical University, Jiangsu, China; 4 Jiangsu Province Engineering Research Center of Stomatological Translational Medicine, Nanjing Medical University, Jiangsu, China

**Keywords:** Head and neck squamous cell carcinoma, Immunogenic cell death, Prognostic model, Immunotherapy, HSP90AA1

## Abstract

**Purpose:**

Head and neck cancer is the sixth most common type of cancer worldwide, wherein the immune responses are closely associated with disease occurrence, development, and prognosis. Investigation of the role of immunogenic cell death-related genes (ICDGs) in adaptive immune response activation may provide cues into the mechanism underlying the outcome of HNSCC immunotherapy.

**Methods:**

ICDGs expression patterns in HNSCC were analyzed, after which consensus clustering in HNSCC cohort conducted. A 4-gene prognostic model was constructed through LASSO and Cox regression analyses to analyze the prognostic index using the TCGA dataset, followed by validation with two GEO datasets. The distribution of immune cells and the response to immunotherapy were compared between different risk subtypes through multiple algorithms. Moreover, immunohistochemical (IHC) analyses were conducted to validate the prognostic value of HSP90AA1 as a predictor of HNSCC patient prognosis. In vitro assays were performed to further detect the effect of HSP90AA1 in the development of HNSCC.

**Results:**

A novel prognostic index based on four ICDGs was constructed and proved to be useful as an independent factor of HNSCC prognosis. The risk score derived from this model grouped patients into high- and low-risk subtypes, wherein the high-risk subtype had worse survival outcomes and poorer immunotherapy response. IHC analysis validated the applicability of HSP90AA1 as a predictor of prognosis of HNSCC patients. HSP90AA1 expression in tumor cells promotes the progression of HNSCC.

**Conclusions:**

Together, these results highlight a novel four-gene prognostic signature as a valuable tool to assess survival status and prognosis of HNSCC patients.

**Supplementary Information:**

The online version contains supplementary material available at 10.1007/s00432-024-05779-2.

## Introduction

Head and neck squamous cell carcinoma (HNSCC) is the most common malignancy that arises in the head and neck region. Most afflicted patients get diagnosed at an advanced stage and exhibit relatively poor outcomes (Johnson et al. 2020). Current treatment strategies for HNSCC involve combinations of surgery, chemotherapy, or radiotherapy but lack an accurate and effective individualized treatment plan. Given the low survival rate and poor prognosis of patients with advanced HNSCC, there is an imperative need to investigate biomarkers that accurately predict tumor behavior at early stages to facilitate development of effective screening and treatment methods for HNSCC (Leemans et al. 2018). Evidence suggests an important role of the immune system in HNSCC pathogenesis. Therefore, continuous development of cancer immunotherapy and treatments targeting T-cell immune checkpoints might reveal biomarkers for accurate prediction and identification of HNSCC immunotherapy (Trivedi et al. 2021).

Immune responses in the tumor microenvironment (TME) are principally involved in tumor pathogenesis and are thought to determine clinical prognosis of patients afflicted with cancer. Cancer is a disease wherein variations in the genome induce expression of tumor antigens recognized by the immune system, which elicits cellular immune responses (Schreiber et al. 2011; Podlaha et al. 2012). Considering the cellular basis of immunotherapy, invasive immune cells develop a series of complex mechanisms to inhibit the development of malignant tumors, prevent tumor cells from immune escape, and improve effects of anti-tumor responses (Rabinovich et al. 2007; Khong and Restifo 2002; Seager et al. 2024). Immune checkpoint inhibitors (ICIs) are the molecules of coinhibitory signaling pathways that have become one of the most important agents in cancer immunotherapies and serve as first-line treatment agents for various solid and liquid tumors (Pardoll 2012). In particular, ICIs targeting CTLA4 and PD-1, two most clinically relevant immune checkpoints, and T-cell checkpoint inhibitors have fundamentally changed the treatment landscape of many cancers (Carlino et al. 2021). Despite the significant curative effects of ICIs, however, resistance to agents and adverse immune responses in cancer patients pose major challenges to the success of cancer immunotherapy (Motzer et al. 2015; Forde et al. 2018). Therefore, the comprehensive knowledge of immune responses in the tumor immune microenvironment is essential to predict prognosis of cancer patients depending on immune events, to design more potent immunotherapies, and ultimately deliver safer and effective treatments (Bagchi et al. 2021).

Immunogenic cell death (ICD), a type of strictly regulated cell death (RCD) process, activates the adaptive immune response against cancer in an immunocompetent setting (Galluzzi et al. 2018). Studies conducted to explore the concept and significance of ICD have demonstrated that the ability of ICD to initiate adaptive immunity is important for clearance of infectious pathogens and to regulate the cancer-immune balance through anti-tumor immune responses (Pfirschke et al. 2016). In general, adjuvant-like signals known as damage-related molecular patterns (DAMPs) are released during ICD, including high mobility group protein B1 (HMGB1), secretion of ATP, and cell surface-exposed calreticulin (CALR) (Galluzzi et al. 2020; Krysko et al. 2012). Numerous preclinical trials and clinical evidence suggest that ICD induction increases the sensitivity of various tumors to ICIs (Pilones et al. 2020). Thus, the use of the combination of ICD inducers and ICIs is thought to achieve better immunotherapy outcomes and predict prognosis of cancer patients (Carlino et al. 2021; Voorwerk et al. 2019).

The continued research and development of cancer immunotherapy alongside the clinical effectiveness of targeted immune checkpoint drugs have demonstrated the importance of biomarkers and prognostic models in the prediction of tumor immunophenotypes (Economopoulou et al. 2016). Specific biomarkers identified based on high-throughput bioinformatic analysis have a long-term impact on guiding treatment and prognosis of different cancers (Filella and Foj 2018). Although several immune-related biomarkers have been investigated to define the tumor immune microenvironment and guide personalized tumor therapy, studies on ICD-related DAMPs and prognostic models as possible immunotherapeutic targets for HNSCC are scarce (Yao et al. 2024). Therefore, it is important to build a comprehensive and an effective immune-related prediction model to better understand the complex anti-tumor immune response and provide cues for an effective immunotherapy that alleviates the quality of life of cancer patients.

Herein, we comprehensively identified the expression pattern of ICD-related DAMPs between HNSCC patients and normal samples to develop a prognostic model that predicts outcomes of patients with HNSCC. Through bioinformatic analyses of extant datasets and further validation of clinical samples, we constructed a novel DAMP-based risk score signature to evaluate the immune microenvironment, prognosis, and immunotherapy response among HNSCC patients. This work may provide new implications for immunotherapy strategies against HNSCC and reinforce the importance of accurate prognosis and individualized precision treatment.

## Materials and methods

### Datasets

TCGA database was used to download RNA-sequencing data and the corresponding clinicopathological data (TPM format and log conversion) of patients with HNSCC (Tomczak et al. 2015). Samples lacking survival status or those with an overall survival (OS) time < 30 days were excluded. The analysis included 499 tumor samples and 44 normal samples. GSE41643 and GSE65858 were downloaded from GEO and screened with the same inclusion and exclusion criteria as validation columns (Barrett et al. 2013). A total of 367 samples were included. GSE78220, IMvigor, and PRJEB23709 comprised the immunotherapy cohorts where the clinical information included the response of patients to immunotherapy. For survival analysis, 499 patients were enrolled in TCGA cohort, 367 patients in GSE41643 and GSE65858, 27 patients in GSE78220, 348 patients in IMvigor, and 726 patients in PRJEB23709. The combat function of sva program was employed to exclude any batch effect on GSE41643, GSE65858, and TCGA-HNSC, and the corrected cohort was termed as “meta-cohort.” GSE103322 was a single-cell sequencing dataset for HNSC, and its processing pipeline was from the TISCH database. Cell localization immunofluorescence staining and immunohistochemistry (IHC) images were obtained from the HPA database. Somatic mutation and CNV data were extracted from TCGA-HNSC cohort of 506 patients. The Maftools package and its “oncoplot” feature were used to render mutational differences. In addition, 29 immunogenic cell death-related genes (ICDGs) were identified from a relevant reference that could be annotated in the cohort (Xu et al. 2022).

### Consensus clustering

Based on prognostic ICDGs (*P* < 0.05), consensus clustering was performed with the ‘ConcensusClusterPlus’ tool in R package to obtain the number of clusters. Subsequently, the ideal cluster numbers were selected for a *K* value between 2 and 10, and this process was replicated 100 times with pltem = 0.8 to verify the stability of subtypes. PCA was used to investigate if each subtype was relatively independent from the others. Kaplan–Meier curves were produced for evaluation of HNSC patient OS using the log-rank test.

### Functional enrichment analysis

The ‘clusterProfiler’ package in R software was used to conduct Gene Ontology (GO) and Kyoto Encyclopedia of Genes and Genomes (KEGG) analyses. Biological processes, functional pathways, and cellular components of genes were annotated among the ICD low and high cohorts. DEGs among different subtypes were investigated using the limma package (P < 0.001) (Yu et al. 2012). Values of P < 0.05 and q < 0.05 indicated significantly enrichment pathways. Gene set enrichment analysis (GSEA) was performed to assess differences in biological pathways between subtypes using c2.cp.kegg.v7.0.symbols.gmt as a reference set and FDR < 0.05 as a screening threshold.

### Characterization of the immune landscape

For identification of the immune characteristics of HNSCC, algorithms such as TIMER, CIBERSORT, MCP-counter, XCELL, QUANTISEQ, and EPIC were simultaneously employed for estimation of abundance of immune cells in different samples. Further, the ESTIMATE algorithm was exploited for calculating immune and interstitial scores to reflect the microenvironment status. Thorsson et al. defined six immunoexpression signature subtypes as per the gene expression profile of whole solid tumors in TCGA, including Wound Healing (Immune C1), IFN-gamma Dominant (Immune C2), Inflammatory (Immune C3), Lymphocyte Depleted (Immune C4), Immunologically Quiet (Immune C5), and TGF-beta Dominant (Immune C6) (Thorsson et al. 2018). We compared the known subtypes with our predicted risk subtypes.

### Construction of an ICD-related risk signature

TCGA-HNSC queue was used for modeling, and GEO queue was employed for external verification. In TCGA-HNSC, redundant genes from ICDGs were removed using the least absolute contraction and selection operator (LASSO) model. A risk score formula was obtained using multivariate Cox regression analysis of integration coefficients and gene expression values. HNSC patients were grouped into high-risk and low-risk subtypes based on the median value of the risk score. Univariate and multivariate Cox regression analyses were performed to determine the prognostic value of the risk score across the entire dataset as well as in the external validation dataset. The prediction accuracy of the risk score was compared with traditional clinicopathological parameters using a time-dependent ROC curve. The survivalROC package was employed to draw an ROC curve and calculate the area under the curve (AUC). Multivariate Cox regression was used to evaluate independent prognostic factors of various clinical indicators; the above factors were combined to construct a prognostic nomogram using the replot package; a calibration curve was used for verification.

### Drug sensitivity analysis

Chemotherapeutics were obtained from the Genomics of Drug Sensitivity in Cancer (GDSC) database. Their IC50 values were determined using a prophetic package in R software.

### IHC staining

*HSP90AA1* expression in HNSCC was validated using a tissue microarray of HNSCC specimens. IHC was conducted as previously described (Zhu et al. 2022). Formalin-fixed, paraffin-embedded HNSCC tissue sections were treated with xylene (deparaffinization) and graded ethanol solutions (rehydration), and then exposed to 3% hydrogen peroxide for 20 min. The samples were blocked and probed for overnight with a rabbit anti-HSP90AA1 antibody (1:30, Santa Cruz Biotechnology, USA). The samples were then treated with HRP-polymer anti-rabbit kit and DAB detection kit (Fuzhou Maixin Biotech, Fuzhou, China).

### IFN-γ analysis by enzyme-linked immunosorbent assay (ELISA)

IFN-γ levels in cell-free media were measured using a human IFN-γ ELISA kit (R&D system, #DY285–05, Minneapolis, MN, USA), as per the manufacturer’s protocol.

### Co-culture assay

Co-culture experiments were conducted using a Transwell co-culture system in 24-well plates with 0.4 μm pore-size insets (Corning Costar, Corning, NY, USA). HN6 and Cal27 cell lines were seeded at a density of 2 × 10^5^ cells/well and cultured in the outer wells in DMEM supplemented with 10% FBS for 24 h. HN6 and Cal27 cell lines were transfected with an *HSP90AA1* knock-down small-interfering RNA or negative control (NC) vector. After 24 h, the medium was removed and the cells were incubated for another 24 h in fresh RPMI medium containing 10% FBS and 1% penicillin/streptomycin. Jurkat cells (4 × 10^5^) were seeded into the upper chamber of the Transwell system. After incubation, the medium was harvested for IFN-γ analysis.

### Cell migration and invasion assays

Wound healing and Transwell assays were performed to detect cell migration and invasion ability in vitro. In the wound healing assay, cells were plated in six-well plates and grown to 90% confuence. A 10 µl pipette tip was used to make a scratch wound into Cal27 and HN6 cell monolayers, and then the cells were incubated in complete medium. Images of the same area of the wound were taken at 0, 12, 18 and 24 h to determine the wound closure rate. Cell invasion assays were performed using Transwell inserts, and the Transwell inserts with the porous membrane (pore size 8 μm, Miliipore), which has been coated with matrigel (Corning, NY, USA) at 37 °C. Approximately 1 × 10^5^ HN6 cells or 2 × 10^5^ Cal27 cells were seeded into the upper chambers and incubated with complete medium in the lower chamber. After 24–36 h, cells were fixed with 4% paraformaldehyde and stained with 0.1% crystal violet (Beyotime, China). Every chamber were counted in 5 randomly selected fields.

### Western blot assay

Western blot assays were performed according to the common methods. Briefy, protein samples were separated using sodium dodecyl sulfate polyacrylamide gel electrophoresis (SDS-PAGE) and transferred to polyvinylidene fuoride (PVDF) membranes (Millipore, Billerica, MA, USA). The membranes were blocked with 5% skimmed milk at room temperature for 2 h and incubated at 4 °C overnight with primary antibodies against GAPDH as a control (1:8000; Proteintech), HSP90AA1 (1:1000; Proteintech), E-cadherin (1:1000; CST), N-cadherin (1:1000; CST), vimentin (1:1000; CST), Snail (1:1000; CST), cleaved caspase 3 (1:1000, CST), caspase 3 (1:1000, Proteintech), BAX (1:1000, Proteintech), BCL-2 (1:1000, Proteintech) followed by incubation with secondary antibodies for 1 h at room temperature. The protein bands were imaged using Tanon High-sig ECL Western Blotting Substrate (180–5001; Tanon).

### Statistical analyses

Kruskal–Wallis test was used to compare differences between groups, and the χ2 test was used for analysis of associations between categorical covariates. A two-sided Student’s t-test was employed to compare two groups. P < 0.05 indicated a statistically significant difference.

## Result

### Landscape of ICDGs in HNSCC

**Fig. 1:**
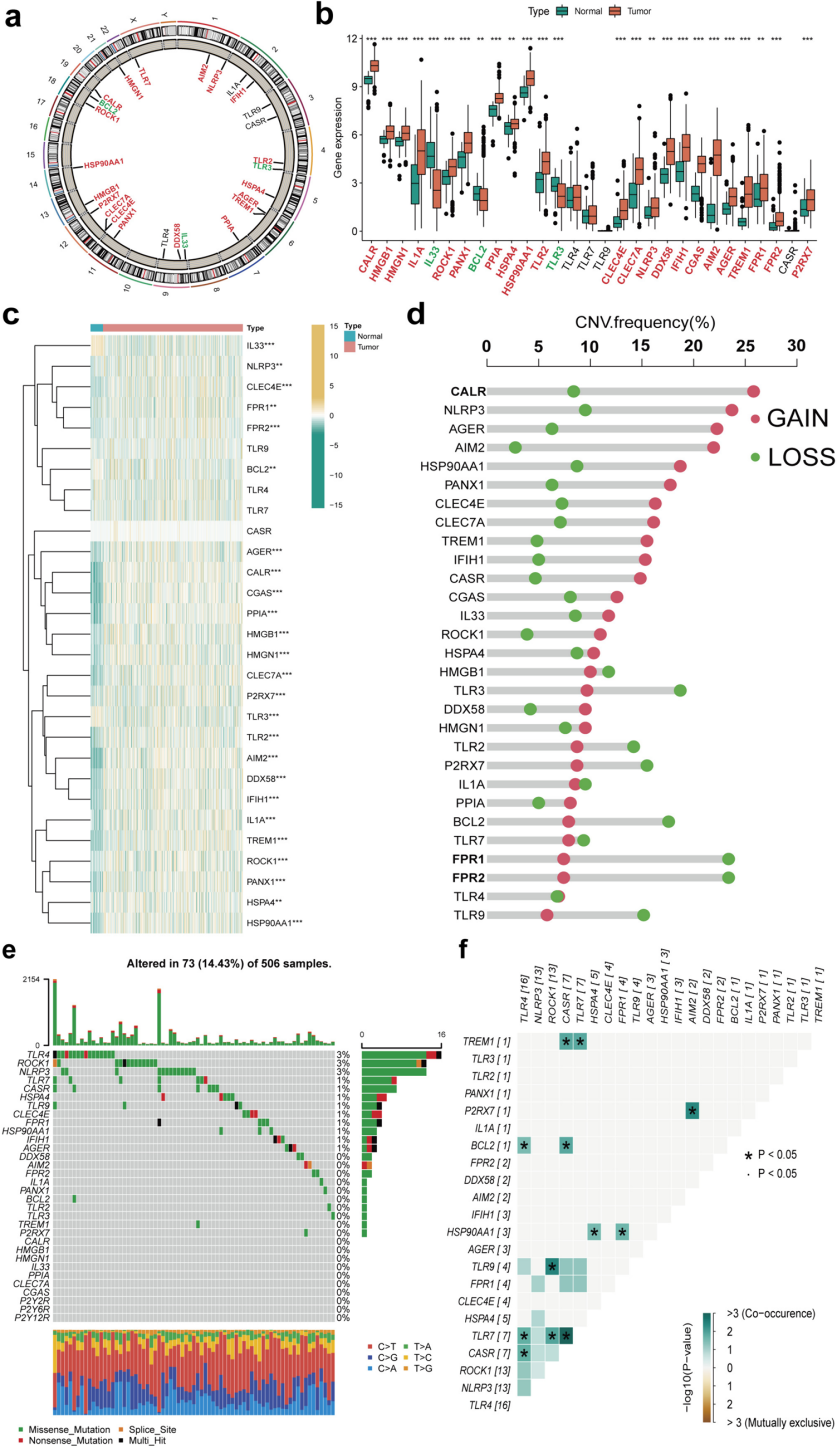
Landscape of the genetic and expression variation of ICDGs in HNSCC. **a** The location of ICDGs on chromosomes in TCGA cohort. **b** Box plot showed the differentially expressed ICDGs between normal or HNSCC patients in TCGA cohort (green: low expression level; red: high expression level). **c** Heatmap of the ICDGs expression (yellow: high expression level; green: low expression level). **d** The CNV frequency of 29 ICDGs in the HNSCC cohort. The length of the column represented the CNV frequency. **e** The mutation frequency of ICDGs in 506 HNSCC patients. **f** The co-occurrence of mutated ICDGs among HNSCC patients in TCGA cohort. The data was presented as mean ± SD, *p < 0.05, **p < 0.01, ***p < 0.001

**Fig. 2:**
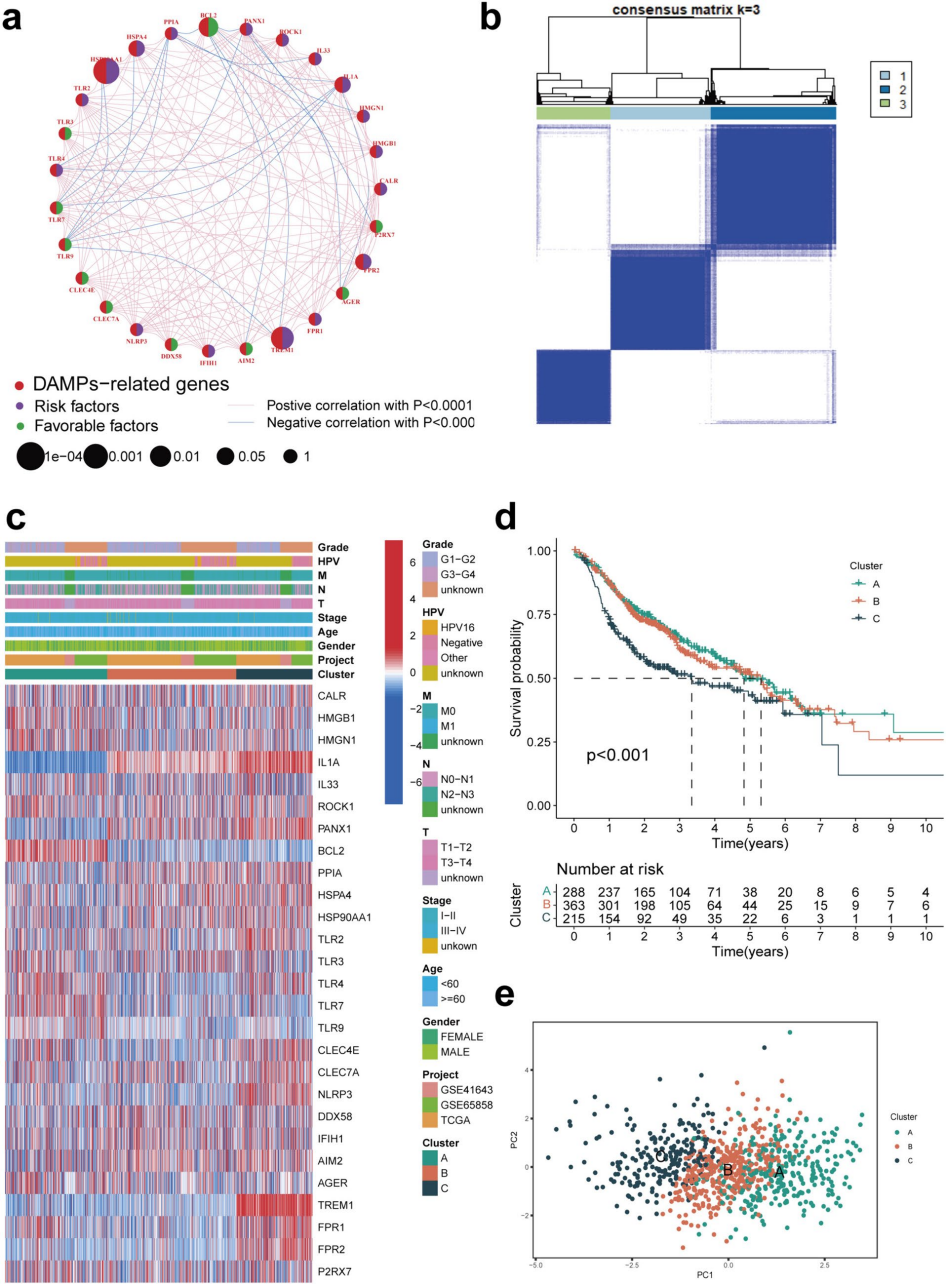
Identification of molecular subtypes based on ICDGs. **a** Co-expression network plot indicated the expression association (blue lines: negative correlation; pink lines: positive correlation) and hazard ratio (purple circle: risk genes; green circle: protective genes) of DAMPs-related genes (ICDGs, indicated as red). **b** 866 HNSCC patients from meta cohort were grouped into three clusters according to the consensus clustering matrix (*k* = 3). **c** Heatmap of the clinicopathologic characters of the three clusters classified by these genes. **d** Kaplan–Meier OS curves for the three clusters. **e** Principal conponents analysis (PCA) of the three clusters

In this study, we identified 29 ICDGs in TCGA-HNSCC dataset; their locations on chromosomes are shown in Fig. 1a. We investigated ICDG mRNA expression in normal and HNSCC samples. Except for *TLR4*, *TLR7*, *TLR9*, and *CASR*, all other ICDGs exhibited significant variations in their expression patterns among different samples (Fig. 1b, c). These ICDGs showed CNV changes, wherein the amplification frequency was maximum for *CALR* and the deletion frequency was maximum for *FPR1* and *FPR2* (Fig. 1d). Of 506 patients from TCGA-HNSCC cohort, 73 had ICDG mutations and the highest mutation frequency was noted for *TLR4* (Fig. 1e). Further analysis revealed the remarkable co-mutations between *TLR7* and *TLR4*, *ROCK*, and *CASR* (Fig. 1f). The above analysis demonstrates the high heterogeneity in the expression of ICDGs among normal and HNSCC samples, which indicated that the imbalance in ICDG expression might critically determine the occurrence and progression of HNSCC. Next, we combined GSE41643, GSE65858, and TCGA-HNSCC datasets for 866 HNSCC patients into a meta-cohort for batch normalization (Fig. S1). The clinical characteristics of patients in these study groups were shown in Table S1. Kaplan–Meier and log-rank test results (Fig. S2) revealed the prognostic value of 20 ICDGs for prediction of HNSCC patient outcomes in accordance with the best cut-off point for each group. The DAMP-related gene network map provided a comprehensive and specific description of the interactions between these genes and demonstrated their prognostic significance (Fig. 2a). Most of these ICDGs exhibited significant interaction associations and could serve as potential prognostic indicators.

### Molecular subtype identification based on ICDGs

To exclude the relationship of ICDG expression with HNSCC, we performed consensus clustering of 866 HNSCC patients included in the meta-cohort. The patients were grouped into three subtypes depending on the expression of ICDGs by identifying the optimal clustering stability at K = 3 (Fig. 2b). Accordingly, 288 patients were grouped into Cluster A, 363 patients in Cluster B, and 215 patients were grouped in Cluster C. The heat map explained the distribution of clinical characteristics of various clusters; most genes analyzed herein exhibited significant overexpression in Cluster A and Cluster C (Fig. 2c). The OS analysis revealed the significant survival disadvantage among three molecular subtypes of HNSCC patients (Fig. 2d). In the PCA, three molecular subtypes were relatively discrete (Fig. 2e).

### Analysis of the immune microenvironment and biological pathways in different HNSCC subtypes

**Fig. 3:**
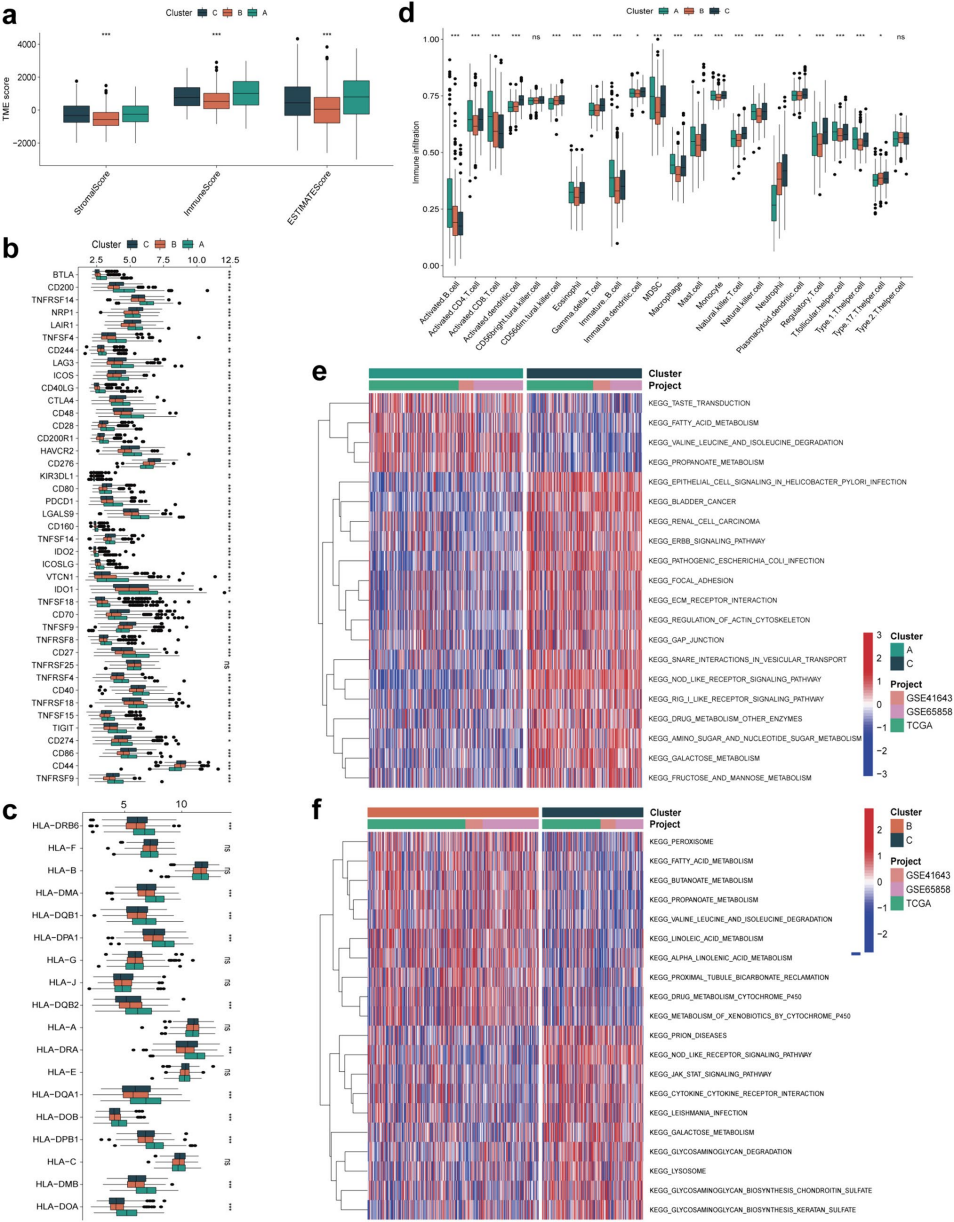
Immune microenvironment and biological pathways in molecular subtypes. **a** Box plot indicated the ESTIMATE score among three clusters. **b** Comparison of the enrichment scores of 41 types of immune cells inhibitors-related genes among three clusters. **c** Comparison of the enrichment scores of HLA-related genes among three clusters. **d** Box plot showed the calculated immune cells infiltration status among three clusters based on GSEA (Light green boxes: cluster A; orange boxes: cluster B; bottle green boxes: cluster C). Heat map showed the GSEA results of the indicated KEGG pathways enrichment between **e** cluster A and C, **f** cluster B and C in the meta cohorts (GSE41643, GSE65858 and TCGA cohort). The data was presented as mean ± SD, *p < 0.05, ***p < 0.001

We employed the ESTIMATE algorithm for calculating the immune status of HNSCC patients and found that Cluster A (previously identified with better patient outcomes in Fig. 2d) exhibited a higher immune score than Cluster B and C (Fig. 3a). In addition, the expression levels of HLA molecules and *ICI* mRNA were reported in different HNSCC molecular subtypes. Interestingly, Cluster A showed higher expression of most ICI mRNAs, including *CD27*, *TNFSF4*, and *TNFRSF14* (Fig. 3b) and most HLA mRNAs such as *HLA-DQA1*, *HLA-J*, *HLA-G*, and *HLA-DQB2* than Cluster B and C (Fig. 3c). The TME status among various molecular subtypes was investigated using GSEA and Cluster A was more likely to exhibit the characteristics of a “hot tumor”, consistent with more immune cell infiltration (Fig. 3d). Therefore, we hypothesized that Cluster A was more sensitive to immunotherapy and would benefit better from immunotherapy treatments. We performed GSEA to explore the biological behavior among different subtypes and found that in comparison with Cluster C, Cluster A showed enrichment of pathways related to TASTE_TRANSDUCTION and FATTY_ACID_METABOLISM (Fig. 3e). In comparison with Cluster C, Cluster B showed enrichment of pathways related to PEROXISOME, FATTY_ACID_METABOLISM, and BUTANOATE_METABOLISM (Fig. 3f). Cluster A exhibited an enriched pathway associated with PRIMARY_IMMUNODEFICIENCY as compared with Cluster B (Fig. S3a). Analysis of the differences between subtypes showed that the 267 DEGs identified in Fig. S3b were mainly associated with IL-17 signaling, cytokine and cytokine receptor interaction, and PI3K-Akt signaling pathway (Fig. S3c, d), suggestive of the potential value of molecular subtypes in predicting TME conditions.

### Identification of risk score in HNSCC

**Fig. 4:**
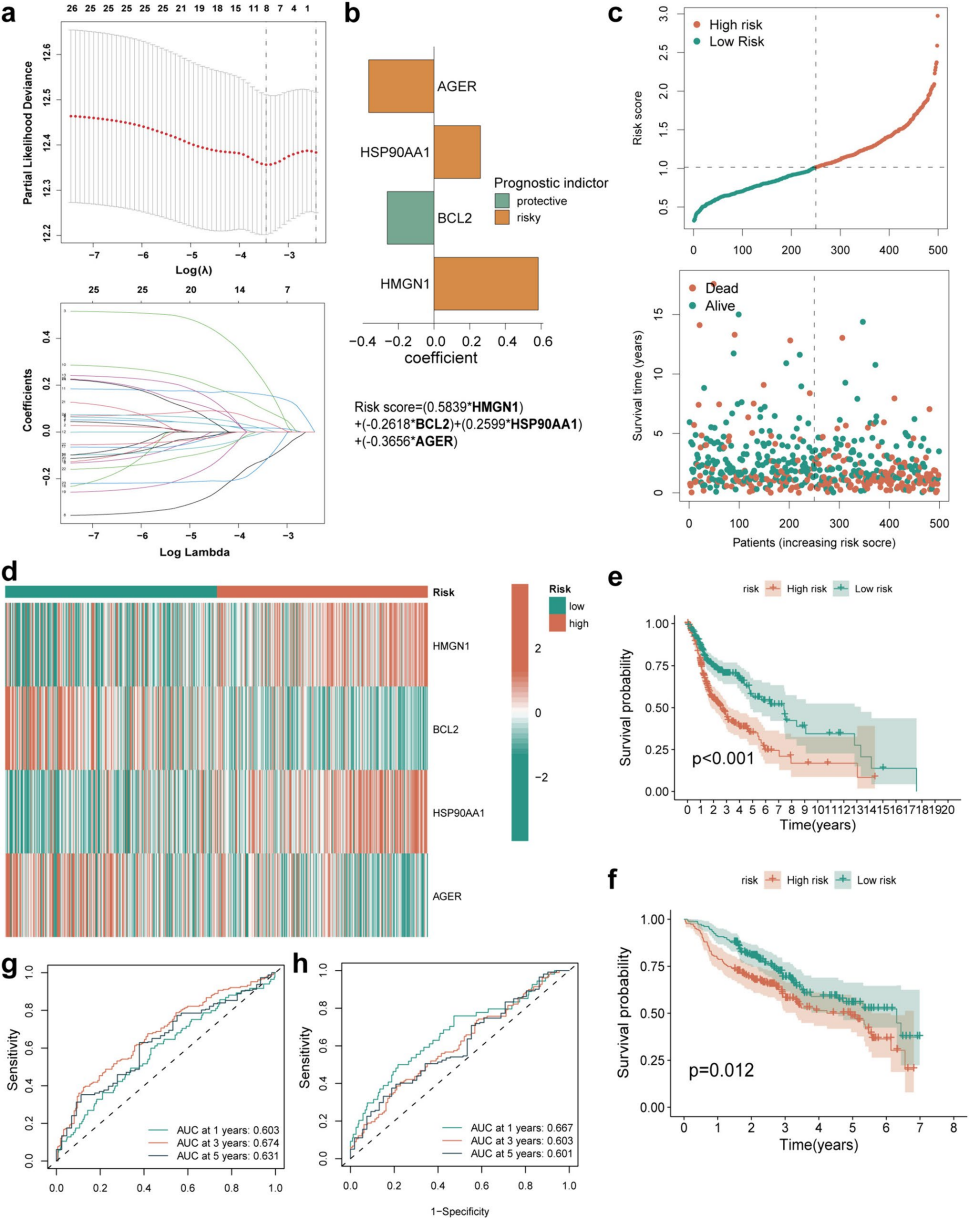
Construction of the risk signature of ICDGs in TCGA cohort. **a** LASSO regression of the OS-related genes followed by cross-validation for tuning the parameter selection in the LASSO regression. **b** Cox regression analysis for constructing the 4-gene prognostic model. **c** The survival status for each patient (low-risk population: on the left side of the dotted line; high-risk population: on the right side of the dotted line) (lower panel). Distribution of patients based on the risk score (upper panel). **d** Heat map indicated the gene expression of the risk model between high- and low-risk group in TCGA cohort. Kaplan–Meier curves for the OS of patients in the high- and low-risk groups in **e** TCGA and **f** GEO cohorts. ROC curves demonstrated the predictive efficiency of the risk score in **g** TCGA and **h** GEO cohorts

**Fig. 5:**
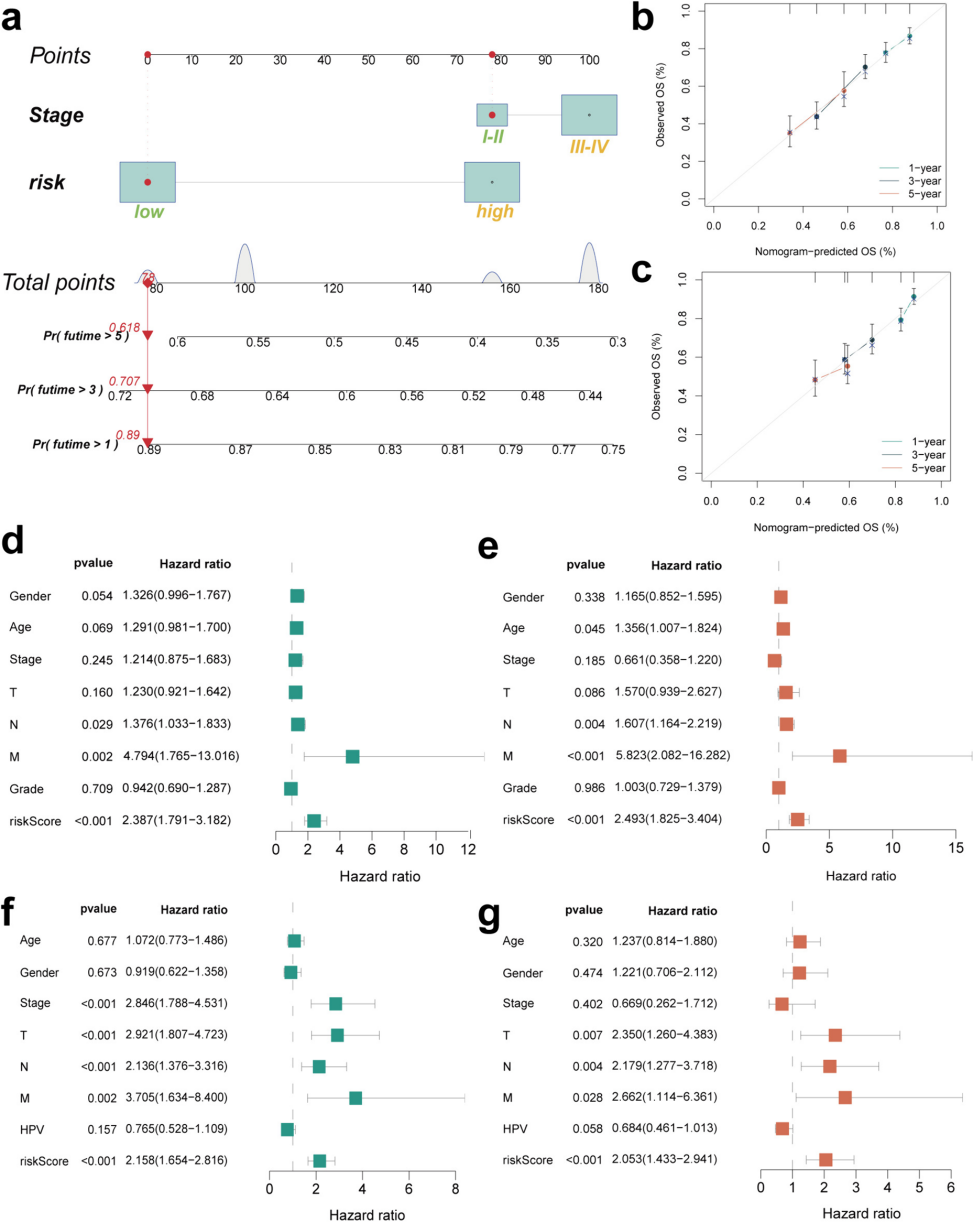
Construction of predictive nomogram and independent prognostic value of risk score. **a**–**c** Nomogram to predict the 1-year, 3-year, and 5-year overall survival rate of HNSCC patients. Calibration curve for the overall survival nomogram model in the discovery group. A dashed diagonal line represents the ideal nomogram. **d**–**e** Hazard ratio and p value of the constituents involved in univariate Cox regression analysis and multivariate analysis considering clinical the parameters and four prognostic ICDGs in TCGA cohort. **f**–**g** Hazard ratio and p value of the constituents involved in univariate Cox regression analysis and multivariate analysis considering clinical the parameters and four prognostic ICDGs in GEO cohort

Next, based on the OS results, we constructed the prognostic model from TCGA cohort. We used the least absolute shrinkage and selection operator (LASSO) model to eliminate redundant genes and obtained coefficients of each gene by multivariate Cox regression (Fig. 4a–b). Ultimately, we constructed a four-gene ICDG signature. For each patient, we estimated the risk score based on the following formula: risk score = (0.5839 × *HMGN1* level) + (− 0.2618 × *BCL2* level) + (0.2599 × *HSP90AA1* level) + (− 0.3656 × *AGER* level). We identified two risk subtypes for HNSCC patients depending on the derived median risk score. There were no significant differences among the clinical characteristics of patients between groups (Table S2). The risk status and survival distribution map revealed the worse prognosis for patients with a higher risk score (Fig. 4c). In TCGA cohort, *HMGN1* and *HSP90AA1* showed significant overexpression in the high-risk subtype and *BCL2* and *AGER* in the low-risk subtypes (Fig. 4d). Survival analysis and 1, 3 and 5-year multi-parameter ROC curves also demonstrated the good prognostic value of our four-gene ICDG signature in both TCGA-HNSCC cohort (Fig. 4e, g, S4a) and the external validation cohort (Fig. 4f, h, S4b). To confirm the clinical application of these results, we constructed a nomogram combining high- and low-risk subtypes with clinical stage of HNSCC (Fig. 5a). Calibration curves obtained demonstrated the good predictive effect of the nanogram in both TCGA-HNSCC cohort (Fig. 5b) and GEO external validation cohort (Fig. 5c). The prediction curve was close to the real curve. Univariate and multivariate Cox regression analyses of routine clinical information revealed this risk score as an independent indicator of HNSCC prognosis (Fig. 5d-g). Together, these observations further validated the accuracy of this model constructed herein for evaluation of HNSCC patient prognosis.

### Association between risk subtypes and therapy among patients with HNSCC

**Fig. 6:**
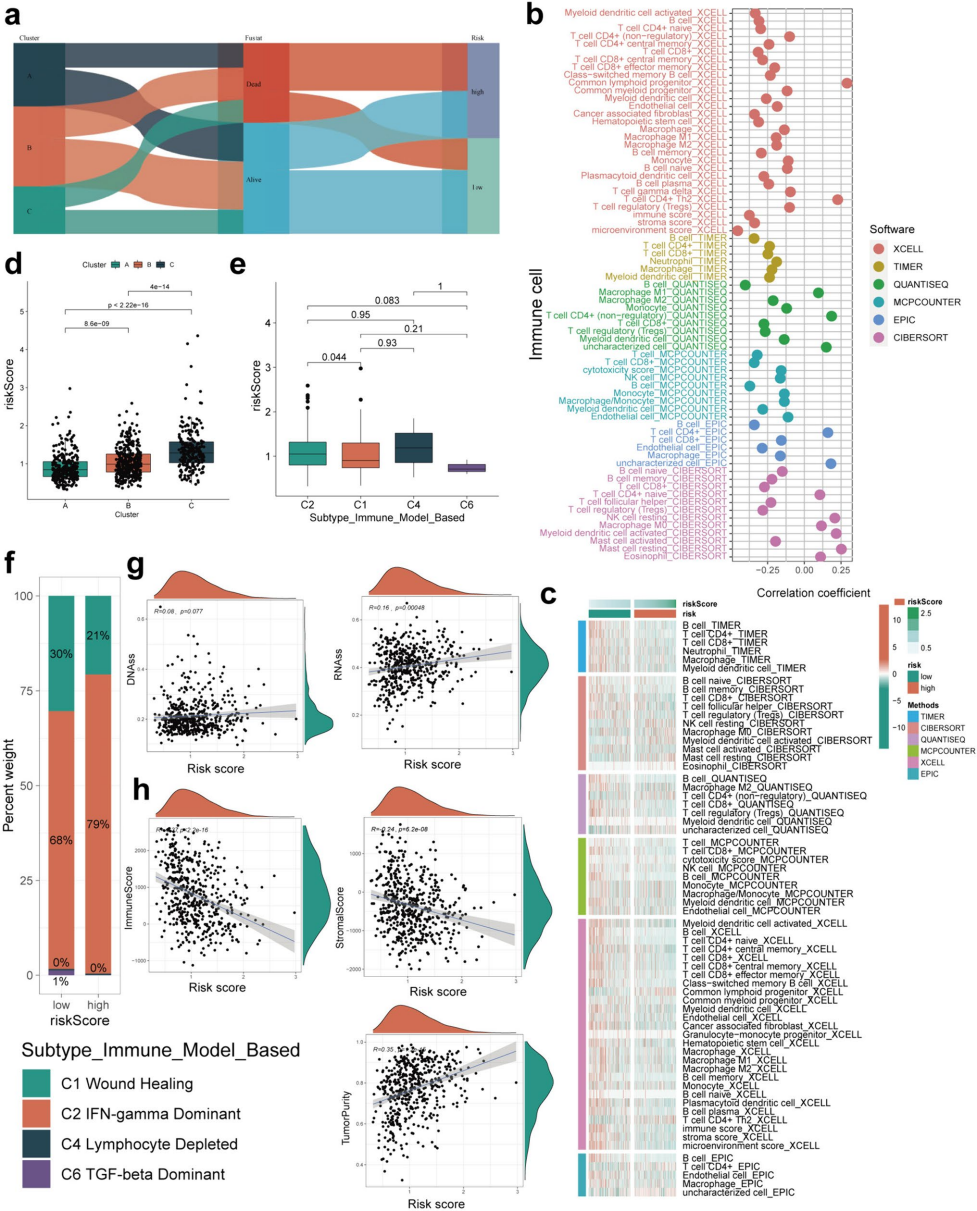
Association between risk subtypes and TME in HNSCC patients. **a** The Sankey diagram showed the potential association between molecular subtypes and risk subtypes in HNSCC patients. **b** The correlation between risk subtypes and immune cell enrichment were calculated by indicated algorithm. **c** Heat map demonstrated the different immune cell distribution between high- and low-risk group. **d** Box plot showed the different risk scores among three clusters of HNSCC patients. **e** The risk scores were calculated in four different immune subtypes (C1, C2, C4, and C6) of HNSCC patients. **f** The propotion of four previously identified immune subtypes of HNSCC patients in high- or low-risk group. The correlation matrix between risk scores and **g** DNAss and RNAss, **h** Immune score, Stroma score and Tumor purity

**Fig. 7:**
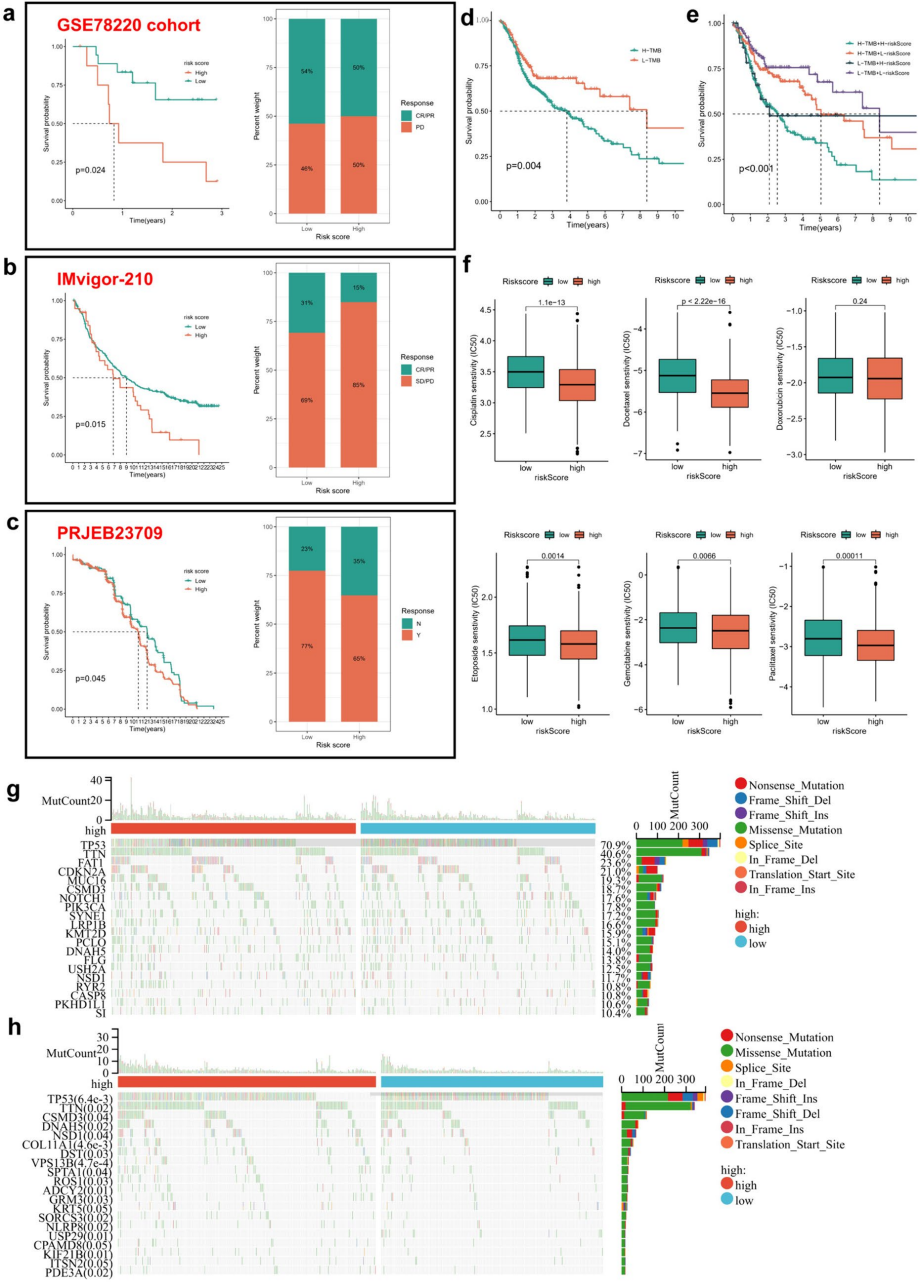
The ICDGs-related risk score was associated with the immune therapy of HNSCC patients. **a**–**c** The Kaplan–Meier curves and immunotherapy response of patients in the high- and low-risk groups from three immunotherapy cohorts (GSE78220, Imvigor-210 and PRJEB23709) were calculated. **d** The Kaplan–Meier curves showed the association of high- or low-tumor mutation burden and OS in HNSCC patients. **e** The OS curves of high- and low-risk HNSCC patients with high or low tumor mutation burden were calculated by Kaplan–Meier. **f** The box plots showed the associations between risk scores and tumor cell sensitivity of different chemotherapeutic drugs. **g**–**h** The landscape of somatic cell mutation status were calculated in high- and low-risk groups (**g**: The top 20 mutated genes; **h**: The top 20 mutated genes with the statistical differences)

The Sankey diagram revealed the close association between the risk subtype and the molecular subtype; most patients within the molecular Cluster A and low-risk subtype were in a viable state (Fig. 6a). We used different algorithms such as TIMER, CIBERSORT, XCELL, QUANTISEQ, MCP-counter, and EPIC for estimation of immune cell abundance in different samples. As expected, the patients with higher risk scores had significant association with the decreased enrichment of CD4^+^T and CD8^+^T cells (Fig. 6b). We also discovered the differences in immune cell enrichment in different risk subtypes (Fig. 6c), consistent with the decrease in immune cell infiltration (CD4^+^T, CD8^+^T, and B cells) observed for the high-risk group. We further explored the association between molecular subtypes and risk subtypes and noticed that Cluster A exhibited the lowest risk score (Fig. 6d). In comparison with the previously identified immune subtypes (Thorsson et al. 2018), the C6 subtype (TGF-beta Dominant) had the lowest risk score and the C4 subtype (Lymphocyte Depleted) had the highest risk score (Fig. 6e); the low-risk subtype had a higher proportion of C1 subtype (Fig. 6f). Given the important influence of the stemness index on the outcomes of immunotherapy, we performed correlation analysis and found that RNAss increased with an increase in the risk score and DNAss showed no statistical significance (Fig. 6g). Similarly, the ESTIMATE algorithm indicated an increase in the tumor purity score and a decrease in the immune score with an increase in the risk score (Fig. 6h), suggesting that the immune activation status in the low-risk subtype may be attributed to the benefit from immunotherapy. To verify the significance of the risk score in the prediction of patient survival and treatment response, we conducted validation in different immunotherapy cohorts (Fig. 7a-c). Our observations suggest that the risk score had a risk stratification value in different immunotherapy cohorts. Low-risk patients had better prognosis and might display better response to immunotherapy. As tumor mutational burden (TMB) determines the outcomes of immunotherapy, we found that the level of TMB itself had a risk stratification effect (Fig. 7d). The patients with low-TMB and low-risk status exhibited the best survival outcomes (Fig. 7e). The IC50 value was estimated for several common chemotherapy drugs, and the majority of patients from the high-risk group were more sensitive to the tested drugs (Fig. 7f). The landscape of somatic cell mutation status in high- and low-risk groups is shown in Fig. 7g, h.

### Expression landscape of four ICDGs

**Fig. 8:**
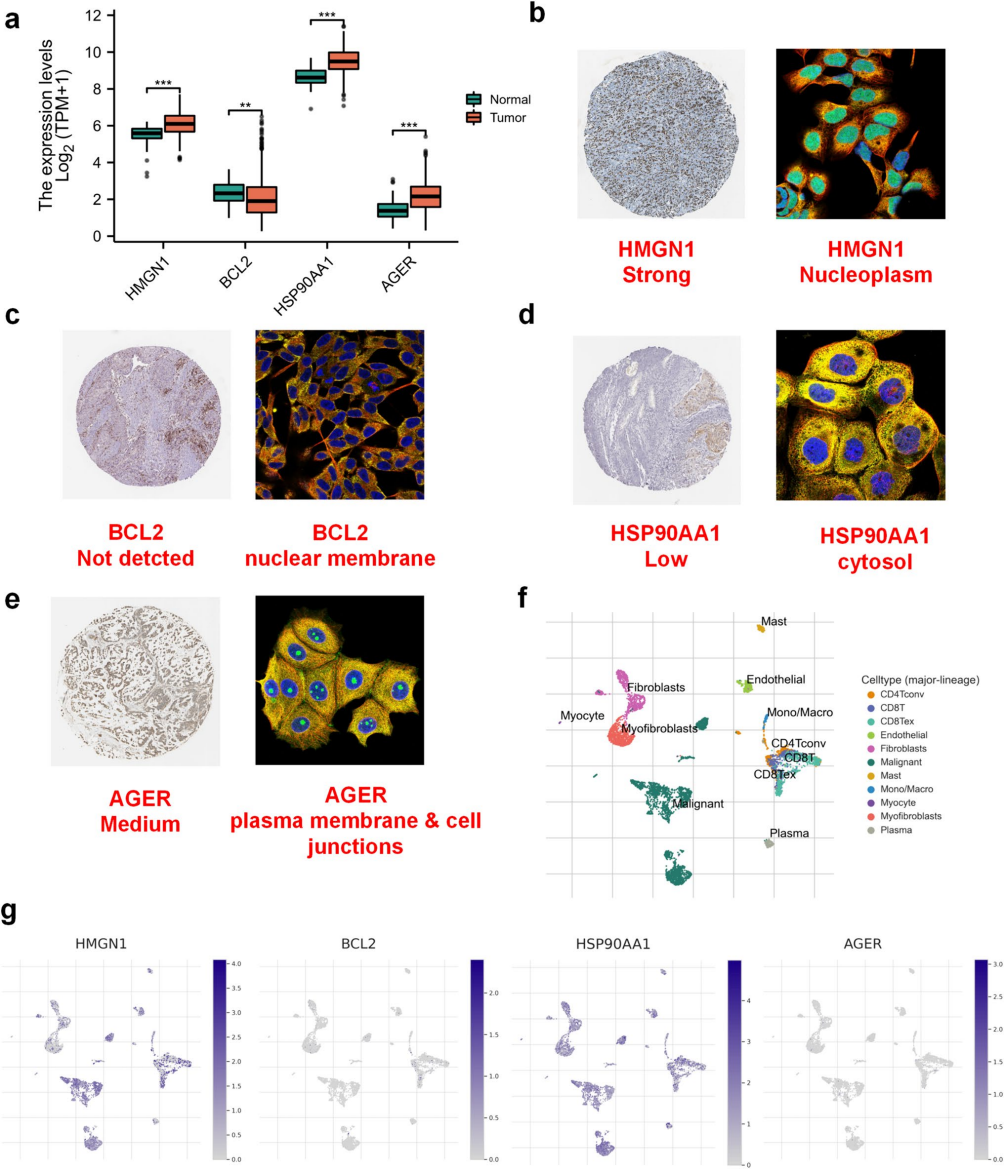
The cellular expression landscape of 4 ICDGs. **a** The gene expression levels of HMGN1, BCL2, HSP90AA1 and AGER in TCGA and GTEx cohorts were calculated. **b**–**e** The relative expression abundance in tumor tissue and subcellular location of 4 ICDGs were calculated by The Human Protein Atlas (HPA). **f**–**g** The relative expression abundance of four ICDGs among different types of the cells were calculated by Tumor Immune Single-Cell Hub (TISCH) dataset

The expression of *HMGN1*, *HSP90AA1*, *BCL2*, and *AGER* was verified in TCGA and GTEx databases, and *HMGN1*, *HSP90AA1* and *AGER* were found to be highly expressed in tumor tissues (Fig. 8a). Sub-localized immunofluorescence analysis and IHC images revealed the expression distribution and staining intensity of different genes (Fig. 8b-e). Single-cell data set showed the significant distribution of *HMGN1* and *HSP90AA1*, but not *BCL2* and *AGER*, in various subsets of cells (Fig. 8f, g). It was worth noting that the survival indicator effects of *HMGN1*, *HSP90AA1*, *BCL2*, and *AGER* were analyzed in multiple cohorts. *HSP90AA1* was a significant survival indicator in multiple cohorts and might represent a risk factor (Fig. S5).

### Increased tumoral *HSP90AA1* expression was associated with poor patient outcomes and impaired T cell function

**Fig. 9:**
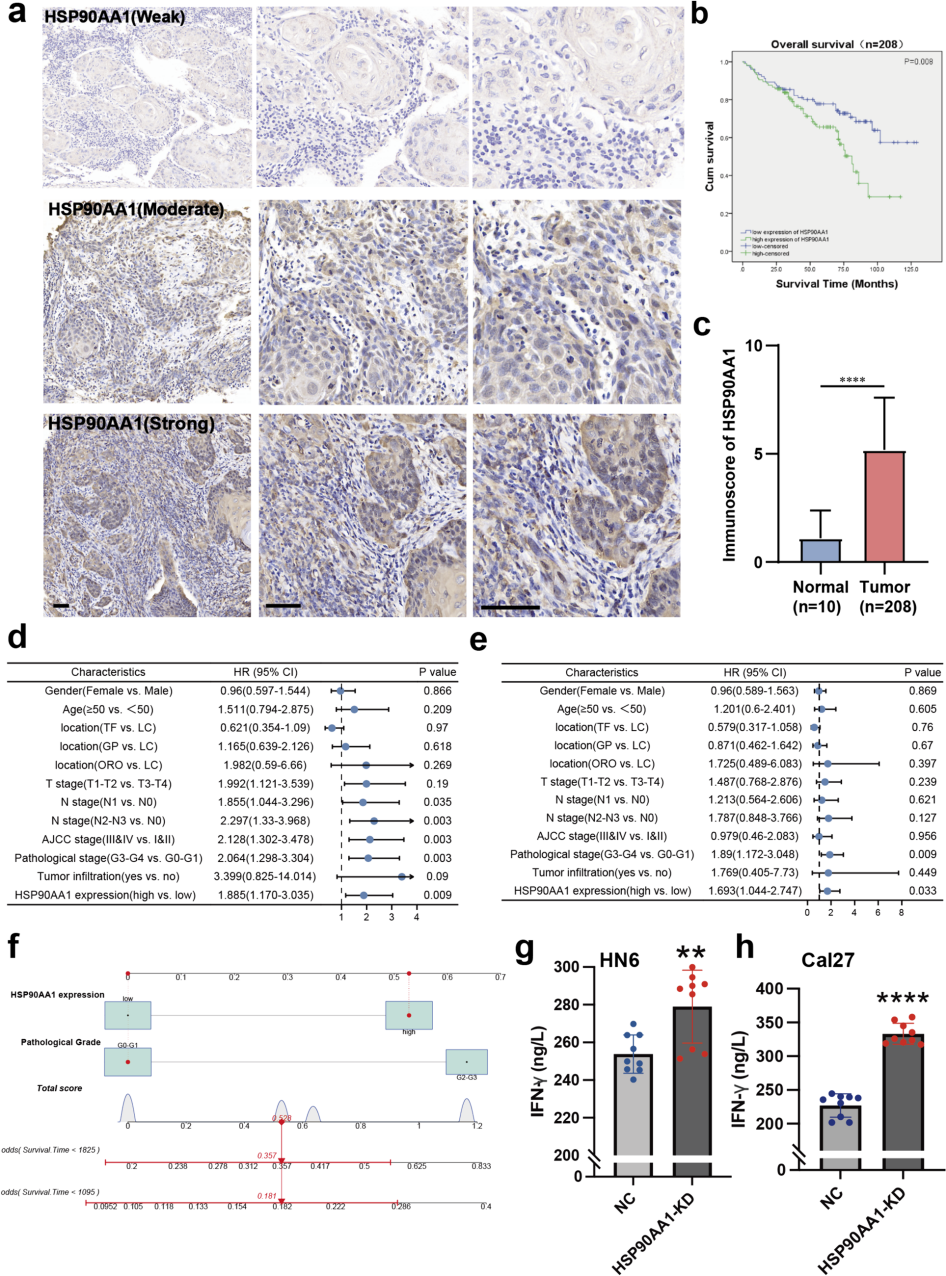
Increased tumoral HSP90AA1 expression was associated with the poor patient outcomes and impaired T cell function. The tissue-microarray of 208-HNSCC patient cohort was subjected to IHC for HSP90AA1 detection. **a** Representative images of different expression level of HSP90AA1 in HNSCC patient tissues. **b** OS curves for HNSCC patients with HSP90AA1-low and HSP90AA1-high expression. **c** The expression of HSP90AA1 was significantly elevated in HNSCC tumor tissues (Normal: normal epithelial tissues; Tumor: HNSCC tumor tissues). Hazard ratio and *p* values of the constituents involved in **d** univariate Cox regression analysis and **e** multivariate Cox regression analysis considering the clinical parameters and HSP90AA1 expression in the 208-HNSCC patient cohort (*TF* Tongue and Mouth Floor, *GP* Gingiva and Palate, *LC* Lip and Cheek, *ORO* Oropharynx). **f** Nomogram to predict the 3-year, and 5-year overall survival rate of HNSCC patients. **g**–**h** The transwell co-culture system were adopted to investigate effector T-cell function impairment. Jurkat cells were co-cultured with either NC vector transfected HN6 or Cal27 cells (“Negative Control”) or siRNA transfected HN6 or Cal27 cells (“si-HSP90AA1”). IFN-γ secretion was measured using ELISA. Data was presented as mean ± SD. The dots plot represent the three independent biological replicates and three technical replicates. Statistical significance was calculated using One-way ANOVA with Tukey multiple comparisons test. **p < 0.01, ****p < 0.0001

To verify the prognostic value of *HSP90AA1*, IHC staining was performed on the tissue-microarray of 208 HNSCC patient cohort to detect HSP90AA1 expression (Fig. 9a). Higher *HSP90AA1* expression was associated with poorer OS in HNSCC patients (Fig. 9b). In comparison with normal tissues, HNSCC tumor tissues showed significant overexpression of *HSP90AA1* (Fig. 9c). Univariate and multivariate Cox regression analyses involving the cohort of 208 HNSCC patients revealed *HSP90AA1* expression level as an independent HNSCC prognostic indicator (Fig. 9d, e). The nomogram constructed by combining *HSP90AA1* expression and pathological grade could effectively predict the 3- and 5-year OS of HNSCC patients (Fig. 9f). To examine the effect of *HSP90AA1* on the function of effector T cells, we cultured Jurkat cells and HN6 or Cal27 cells using the Transwell co-culture system. The ability of Jurkat cells to secret IFN-γ was enhanced after co-culture with *HSP90AA1*-knockdown oral cancer cells (Fig. 9g, h). In conclusion, *HSP90AA1* may serve as a survival predictor of HNSCC patients and could have an important role in HNSCC progression by impairing the function of effector T cells.

### *HSP90AA1* promotes the invasion, migration and inhibits apoptosis of HNSCC cell lines

**Fig. 10:**
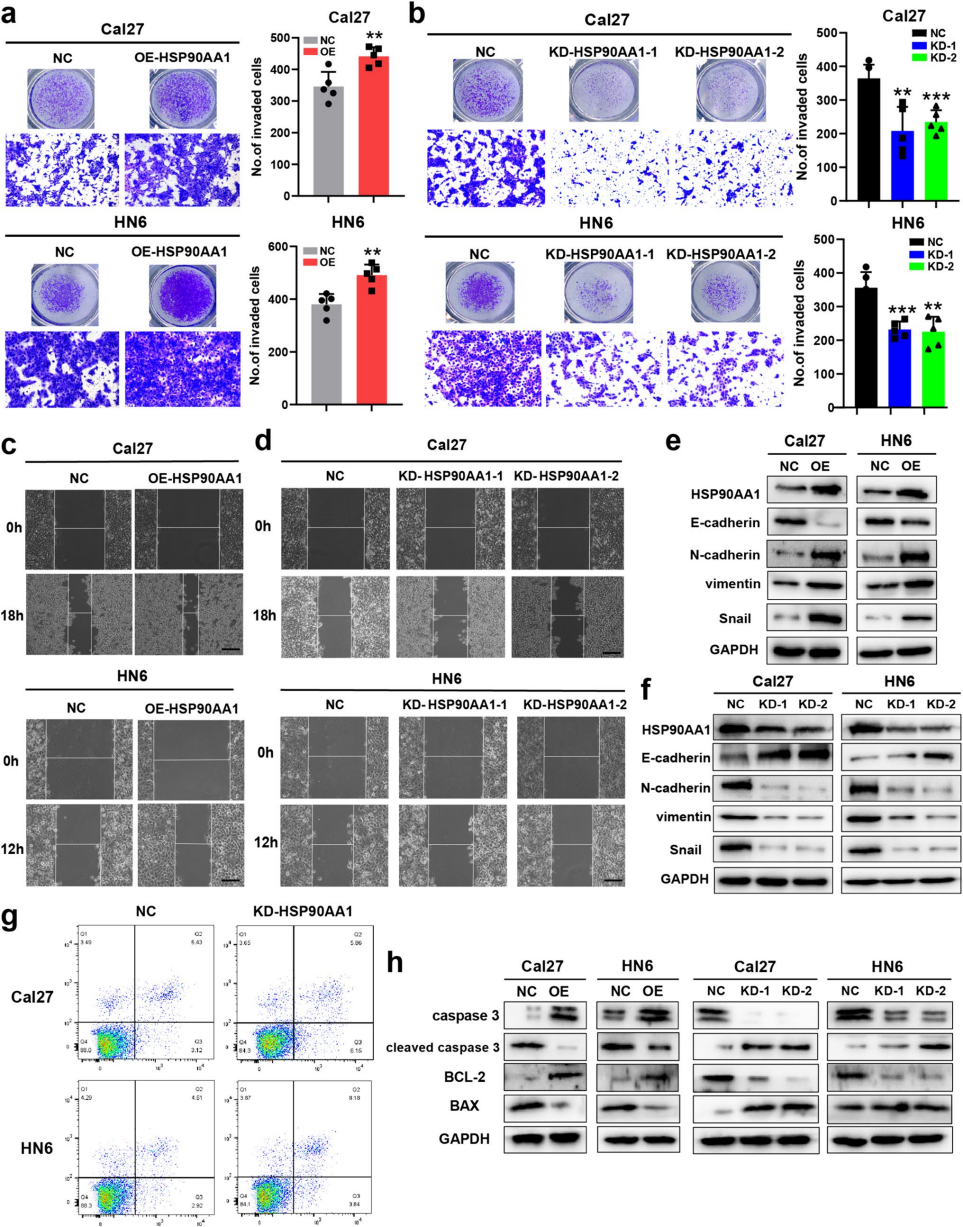
HSP90AA1 promotes the invasion, migration and inhibits apoptosis of HNSCC cells. Small interferon RNAs (si-RNAs) and pcDNA 3.1 plasmids were used to knockdown(KD) or overexpress (OE) the HSP90AA1 expression. **a**–**b** Transwell assays of Cal27 and HN6 cell lines were used to determine the invasion of HNSCC cells. **c**–**d** Wound healing assays of Cal27 and HN6 cell lines were used to determine the migration of HNSCC cells. **e**–**f** The protein expression levels of EMT markers were detected via Western blot analysis in Cal27 and HN6 cell lines. **g** Apoptosis was analyzed by measuring Annexin V-PE/PI positive cells by flow cytometry. **h** Expression levels of apoptosis-related proteins were detected via Western blot analysis in Cal27 and HN6 cell lines. Data was presented as mean ± SD, *P < 0.05, **P < 0.01, ***P < 0.001. Scale bar = 100 μm

To demonstrate the effects of *HSP90AA1* in HNSCC development, we performed cell invasion and migration assays after overexpressing or knocking down *HSP90AA1* in Cal27 and HN6 cell lines. Transwell assays demonstrated that the overexpression of *HSP90AA1* resulted in enhanced cell invasion, whereas *HSP90AA1* knockdown inhibited the invasion of Cal27 and HN6 cells (Fig. 10a, b). Also, wound healing assays revealed that *HSP90AA1* significantly promotes cellular migration (Fig. 10c, d). We further assayed the effects of *HSP90AA1* on EMT-associated indexes via western blot, and found that N-cadherin, vimentin, and Snail in HSP90AA1 knockdown group decreased, but E-cadherin increased; the opposite results were observed in the overexpression of *HSP90AA1* group (Fig. 10e, f). Then, we detected the apoptosis level of HNSCC cell lines. Upon *HSP90AA1* knockdown, the proportions of apoptotic cells were significantly elevated, together with the upregulation of apoptotic markers cleaved-caspase 3, BAX and downregulation of anti-apoptotic protein BCL-2 (Fig. 10g, h). These results indicated *HSP90AA1* inhibited the apoptosis of HNSCC cell lines. Collectively, these results suggested that *HSP90AA1* can act as a tumor promoting factor via promoting the invasion, migration and inhibiting apoptosis of HNSCC cells.

## Discussion

New immunotherapy regimens offer great promises for treatment of different malignancies. However, despite the development of molecular targeted therapies and immunotherapies, the 5-year survival rate among HNSCC patients is low (Li et al. 2020). The secretion of ICD-associated DAMPs and their interactions with innate immune receptors may lead to the activation of the anti-tumor immunity, thus highlighting their role as potential predictors of response to immunotherapy in HNSCC (Krysko et al. 2012). Here, we demonstrated a close association between the expression of ICDGs and HNSCC prognosis and tumor immune microenvironment. We used consensus clustering and identified three subgroups depending on ICDG expression, and revealed differences in clinical outcomes and immune cell infiltration among these clusters. Moreover, we constructed and validated a prognostic risk signature with four ICDGs that stratified our HNSCC patient population into high- and low-risk groups. This risk prediction model played an important role for predicting the OS of HNSCC patients and might act as an independent prognostic indicator.

Various types of cell death processes are tightly regulated by genetic coding mechanisms that target the elimination of irreversibly damaged or potentially harmful cells (Fuchs and Steller 2015; Pasparakis and Vandenabeele 2015). RCD is governed by specific stress effectors and triggers multiple signaling cascades that result in unique functional, immunological, and pathological consequences (Tang et al. 2019). ICD is a unique form of RCD that initiates an adaptive immune response (Galluzzi et al. 2020, 2017). Stress-induced RCD can drive inflammatory responses, activate cytotoxic T lymphocyte (CTL)-mediated adaptive immunity, and establish a long-term immune memory (Galluzzi et al. 2020). There are many different cellular stressors that trigger ICD, including certain chemotherapeutic drugs, therapeutic oncolytic viruses, epigenetic modifiers, numerous physical interventions, and extracorporeal photochemotherapy (Kopecka et al. 2018; Fragale et al. 2017; West et al. 2013; Ventura et al. 2018). Kopecka et al. investigated chemotherapeutic drug resistance in malignant pleural mesothelioma and found that the combination of carfilzomib, chloroquine, and cisplatin enhanced the ER stress-induced apoptosis and ICD in patient samples (Kopecka et al. 2018). Studies have proposed that ICD could turn dying cancer cells into therapeutic vaccines that stimulate anti-tumor immune responses; for example, cisplatin combined with high-dose crizotinib induced ICD in NSCLC cells and effectively controlled the growth of various orthotopic NSCLC (Liu et al. 2019). Therefore, ICD and tumor immunotherapy are closely related, and the roles of ICDGs in determining the prognosis of HNSCC are yet to be investigated. Here, we evaluated expression levels of ICDGs in normal and HNSCC samples in TCGA cohort and found the prognostic values of multiple ICDGs in HNSCC patients. Pathway enrichment analysis demonstrated the involvement of these factors with IL-17 and PI3K-Akt signaling pathways. Consensus clustering allowed us to group HNSCC patients into three molecular subtypes, wherein the subtype with high DAMP expression showed higher immune scores (immune-hot phenotype). Therefore, in-depth investigation of ICDGs may provide novel ideas to strengthen our understanding of occurrence and progression of HNSCC as well as encourage development of anti-tumor immunotherapies.

With the exception of early-stage oral or laryngeal cancer, which can be treated with surgery or radiation alone, most HNSCC patients require the combination of multiple treatments along with multidisciplinary care (Forastiere et al. 2018). The analysis of molecular characteristics and immune components of HNSCC patients highlighted the importance of early diagnosis and screening with predictive biomarkers to overcome the difficulties associated with targeted therapy as well as to prolong survival time and improve quality of prognosis (Johnson et al. 2020). There is an unmet need to develop tumor biomarkers as tools for screening and early detection of tumors, predicting prognosis, and guiding precision therapy. Existing analytical methods, including genomic (DNA), transcriptome (RNA), and nanotechnology, have revealed several biomarkers that have been applied to clinical diagnosis (Hristova and Chan 2019; Sarhadi and Armengol 2022). Next-generation sequencing (NGS) has greatly increased the sensitivity and high throughput capabilities of genomic techniques, and provides technical support for the exploration of tumor biomarkers and their clinical applications (Hristova and Chan 2019). For instance, the mutational status of oncogenes such as *KRAS* and *EGFR* and *ALK* rearrangements have been revealed as predictive indicators of malignant disease, and new drugs such as inhibitors of these molecules have shown improvements in treatment outcomes of advanced NSCLC (Vincent et al. 2012). The analysis of DNA methylome, a common epigenetic phenomenon in cancer, has benefited from the development of high-throughput sequencing technology. Szmida and collaborators revealed that hypermethylation of *PKCB* was significantly associated with *KRAS* mutation, which negatively regulates colon tumor progression (Szmida et al. 2015). Exploration of new cancer biomarkers and risk prediction models with more specific and positive predictive values demands continuous improvement and development in the field of cancer biomarkers. Herein, we constructed a prognostic risk signature with four ICDGs (*HMGN1*, *BCL2*, *HSP90AA1*, *AGER*) by LASSO Cox regression approach, which divided HNSCC patients into two cohorts as per the risk score calculated by this model. Patients with a high-risk score exhibited worse survival outcomes than the patients from the low-risk subgroup, which reiterated the effective prognostic value of our model. The external cohort validation further confirmed the applicability of the risk score for HNSCC prognosis. As a key molecule in mediating anti-tumor immunity, HMGN1 has the potential to be used in combination with checkpoint inhibitors or traditional cancer treatments such as surgery, chemotherapy, and radiotherapy to promote IFN-γ and cytotoxic T lymphocyte production to develop protective immunity against cancers (Yang et al. 2015; Couzin-Frankel 2013). Many members of the BCL2 family that regulate apoptotic pathways are known to exert tumor-suppressive effects (Delbridge and Strasser 2015). Advanced glycosylation end product specific receptor (AGER), which recognizes endogenous molecules released during chronic inflammation, is one of the innate immune pattern recognition receptors abnormally overexpressed in lung cancer (Wang et al. 2015). Therefore, our risk prediction model based on these four genes is of great value in understanding the effect of ICD on the prognosis of HNSCC.

Tumorigenesis and cancer progression are largely associated with the interaction between cancer cells and their TME, especially their immune components (Schreiber et al. 2011; Salmon et al. 2019). The types and locations of immune cells in the TME may predict cancer patient survival status and treatment response (Fridman et al. 2012). Innate immune cells such as natural killer (NK) cells, neutrophils, dendritic cells (DCs), and macrophages inhibit tumor progression through direct apoptosis of tumor cells or by triggering adaptive immune responses. However, cancer cells have developed many approaches to evade immune surveillance, including defects in antigen presentation mechanisms and upregulation in negative regulatory pathways, that lead to a weak anti-tumor immune response (Zhang and Zhang 2020). Cancer immune editing, including all stages of interaction between cancer cells and the immune system except for immune surveillance, comprises three stages, namely, elimination, balance and escape, is the process by which the immune system is limited to promote tumor development (O'Donnell et al. 2019). Immuno-edited tumor cells that have escaped the immune system’s elimination phase undergo sub-clonal differentiation into less immunogenic tumors, which escape the immune surveillance and stimulate modifications such as reduced T cell and IFN-γ production. This phenomenon results in the loss of antigen presentation or reduced PD-L1 expression (Riaz et al. 2017; Takeda et al. 2017). Immune checkpoints that limit immune responses can allow self-tolerance by switching off the functions of cytotoxic T cells to prevent the activated T cell-mediated tissue damage. Thus, these checkpoints may serve as factors that aid cancer cells to evade immune surveillance and may act as probable therapeutic targets (Chhabra and Kennedy 2021). In recent years, inhibitors specifically targeting immune checkpoints, such as CTLA4, PD-1, and PD-L1, have gained popularity in anticancer immunotherapy and have demonstrated clinical efficacy in many cancer types (Sharma and Allison 2015). In our study, multiple algorithms were employed for analysis of the composition of immune cells in the TME. We found variations in the immune cell distribution in each risk subtype. The higher the risk index, the lower was the abundance of cytotoxic immune cells such as CD4^+^ T and CD8^+^ T cells as well as the immune score and tumor purity. Throughout the immune editing process, CD8^+^ and CD4^+^ T cells recognized non-self peptide epitopes that were displayed by MHCI and MHCII molecules expressed on tumor cells, and contributed to the suppression of tumor growth (Linnemann et al. 2015; Schumacher and Schreiber 2015). One of the distinguishing features of human cancer is the increase in the mutation rate. Previous studies have quantified TMB across various cancer types (Chalmers et al. 2017). Tumors with higher TMB were more likely to express immunogenic antigens recognized by T cells and responded to ICIs (O'Donnell et al. 2019; Yarchoan et al. 2017). Thus, we found that there was a risk stratification effect of high and low TMB, and the combination of TMB and risk score offered more advantages in the prediction of survival status. HNSCC patients with high TMB and low-risk score had the best survival outcome. Meanwhile, the efficacy of our risk score in predicting survival outcome and treatment response in HNSCC patients was confirmed in multiple immunotherapy cohorts, suggesting that our ICD-related prediction model can identify the immune status in the TME.

Among the four ICDGs, *HSP90AA1* was highly expressed in tumor tissues and served as a significant survival indicator in multiple cohorts. Previous studies have shown that heat shock protein 90 (HSP90) interacts with multiple pro-tumorigenesis proteins and is an important driver of malignant transformation and progression of tumors. Cancer patients with loss of *HSP90AA1* expression were associated with a good prognosis after surgical treatment, and the absence of Hsp90α in tumor biopsy might serve as an indicator of positive clinical outcome (Buffart et al. 2012; Cheng et al. 2012). *HSP90AA1*, as an important regulator of autophagy, promoted autophagy through the PI3K/Akt/mTOR pathway, inhibited apoptosis through the c-Junction N terminal kinase (JNK)/P38 pathway, and was a critical factor in the development of osteosarcoma chemoresistance (Xiao et al. 2018). Bohonowych and colleagues investigated whether the extracellular heat shock protein 90α (eHSP90α) secreted by tumor cells might trigger a reactive matrix microenvironment and found its role in promoting prostate cancer progression via induction of inflammation through the activation of NF-kB and STAT3, including the transcription and secretion of proinflammatory cytokines IL-6 and IL-8 (Bohonowych et al. 2014). In another study, a nomogram model constructed by database and clinical parameter analyses to evaluate the prognosis and metastasis risk of breast cancer revealed pretreatment plasma HSP90AA1 combined with other biomarkers to easily predict the risk of breast cancer incidence and metastasis (Liu et al. 2021). Similarly, based on the Cox regression analysis, we found that *HSP90AA1* might act as the most significant survival indicator in multiple HNSCC cohorts among four genes included in our risk model. Higher levels of *HSP90AA1* were related to worse prognosis of HNSCC patients in IHC. Cox regression analysis and nomogram also highlighted the potential role of *HSP90AA1* in predicting HNSCC outcomes. T cells are known to shape immune responses in cancer and mediate autoimmunity, such that CD4^+^ T helper (Th) and CD8^+^ T cells mediated effector responses. The functional balance between effector T cells and Treg cells coordinated immune homeostasis and played as an important role in tumorigenesis and progression (Wei et al. 2021). Therefore, we examined the inhibitory effect of *HSP90AA1* expression in tumor cells on IFN-γ secretion by T cells; we confirmed the role of *HSP90AA1* in the immunosuppressive phenotype of HNSCC. In vitro experiments have also highlighted the important role of *HSP90AA1* in promoting the development of HNSCC. Follow-up studies are urgently warranted to further validate the predictive value of *HSP90AA1* and other ICDGs in HNSCC immunotherapy and the anti-tumor immune mechanism.

## Conclusion

In conclusion, we used multi-databases and algorithms to analyze and verify ICDGs that may serve as potential biomarkers for HNSCC prognosis. The risk scores derived from the four ICDG model served as independent predictors of HNSCC patient clinicopathological characteristics and outcomes. Immune and functional analyses revealed the high abundance of immune cells, high TMB, and better response to immunotherapy in the low-risk group. IHC analysis further affirmed the value of *HSP90AA1* as an important HNSCC prognosis predictor. Our study may provide a new perspective and theoretical basis for future research of ICD in HNSCC.

### Supplementary Information

Below is the link to the electronic supplementary material.

Supplementary file1 (DOCX 2233 KB)

Supplementary file2 (XLS 305 KB)

Supplementary file3 (XLS 27 KB)

## Data Availability

The datasets generated during and/or analysed during the current study are available from the corresponding author on reasonable request.
