# Supplementary material for: Identification of immunogenic cell death-related damage-related molecular patterns (DAMPs) to predict outcomes in patients with head and neck squamous cell carcinoma
Source: J Cancer Res Clin Oncol. 2024 May 7;150(5):240. doi: 10.1007/s00432-024-05779-2 (PMC11076381; doi:10.1007/s00432-024-05779-2)
Supplement: Supplementary file 1 — Supplementary file1 (DOCX 2233 KB) [file 432_2024_5779_MOESM1_ESM.docx]

**Supplementary Figures：**

**
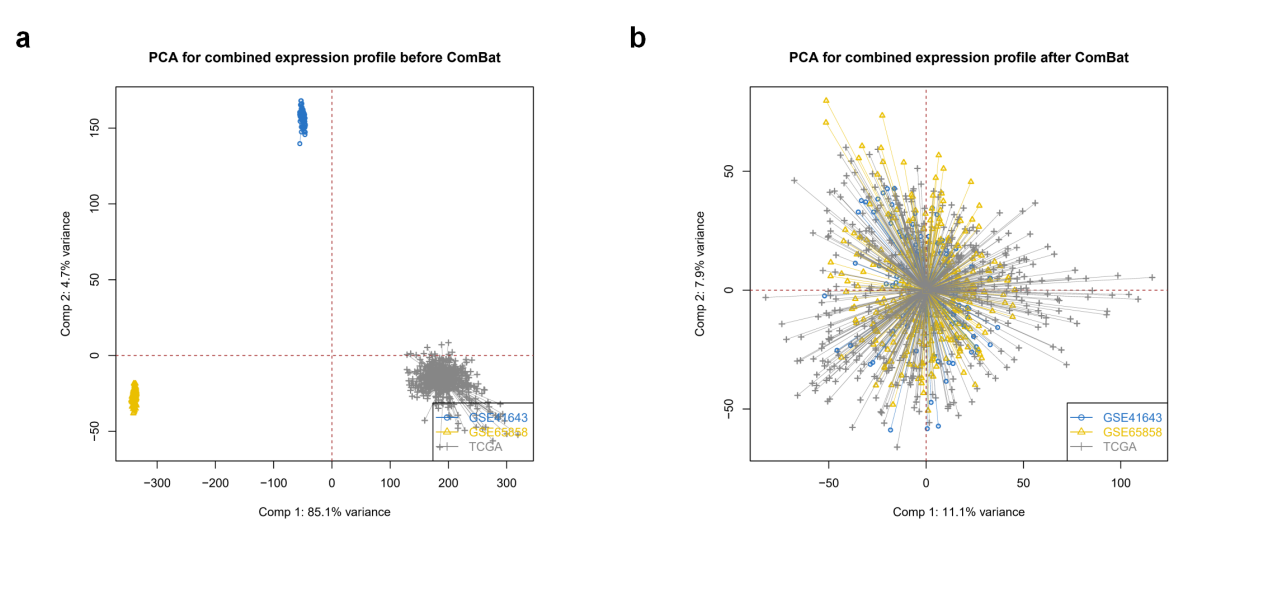
**

**Fig. S1 Batch normalization of combined meta cohort of HNSCC patients.**

(a) PCA for combined expression profile before ComBat. (b) Plot demonstrated the PCA for combined expression profile after ComBat.


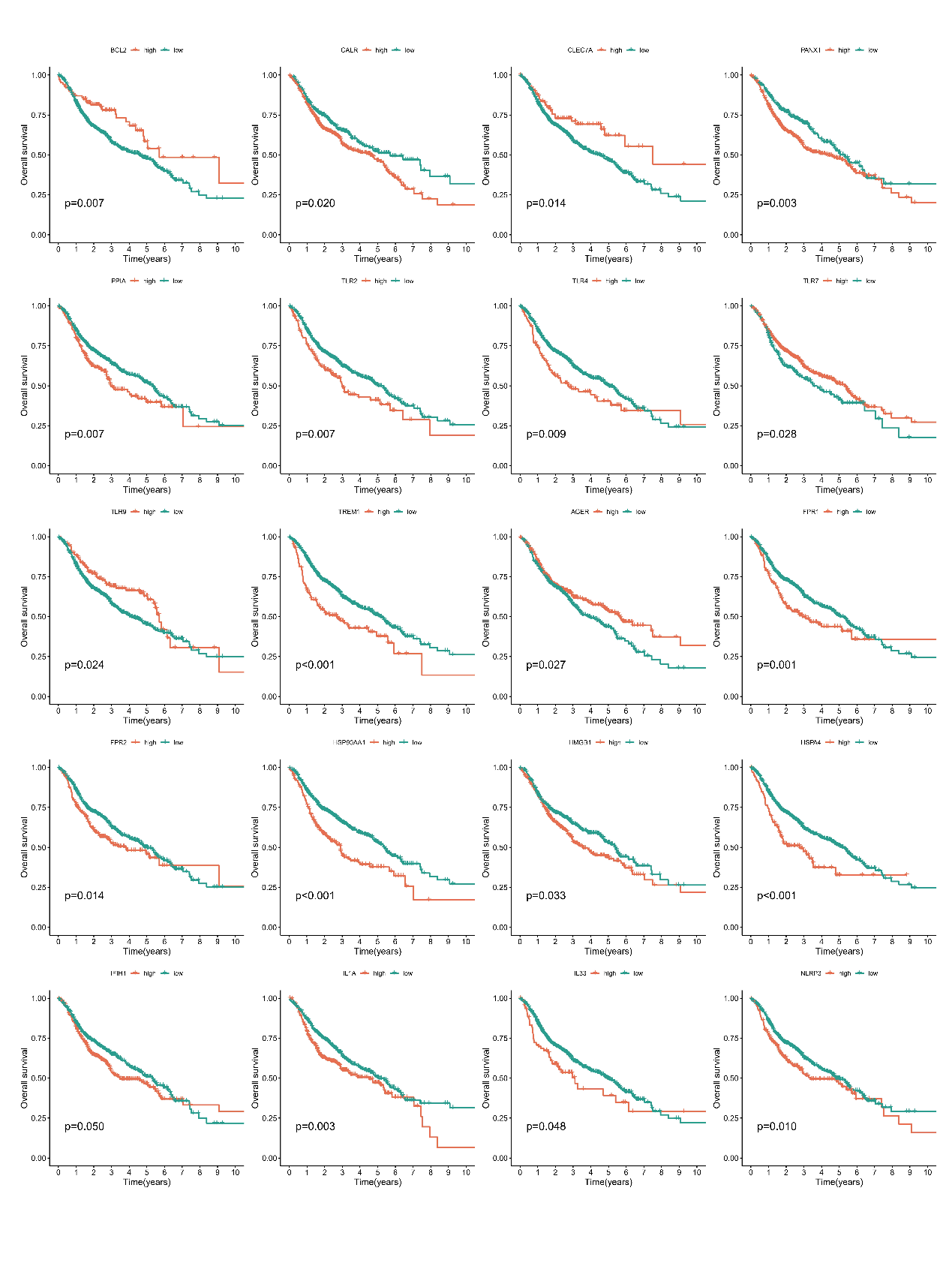


**Fig. S2 The ICDGs were associated with the HNSCC patient outcomes.**

The Kaplan–Meier curves demonstrated the potential value of the 20 prognostic-related ICDGs in predicting HNSCC patient outcomes.

**
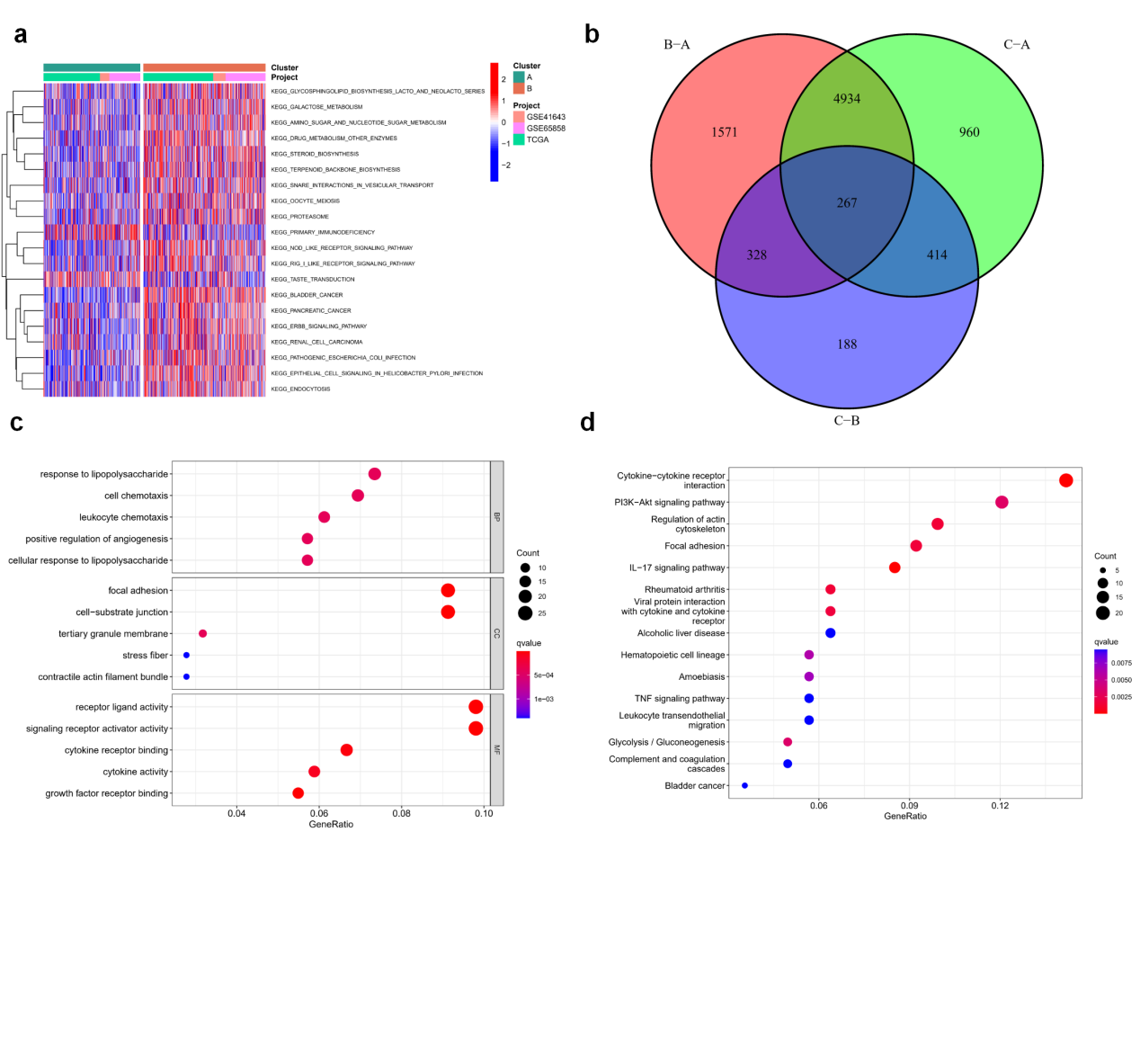
**

**Fig. S3 The biological pathways enrichment in different molecular subtypes.**

(a) Heat map showed the GSEA result of the indicated KEGG pathways enrichment between cluster A and B in the meta cohorts (GSE41643, GSE65858 and TCGA cohort). (b) Venn diagram showed the 267 co-existed genes in three groups. (c) The enriched item of 267 co-existed genes in gene ontology analysis (BP biological process, CC cellular component, MF molecular function). (d) The enriched item of these genes in Kyoto Encyclopedia of Genes and Genomes analysis.


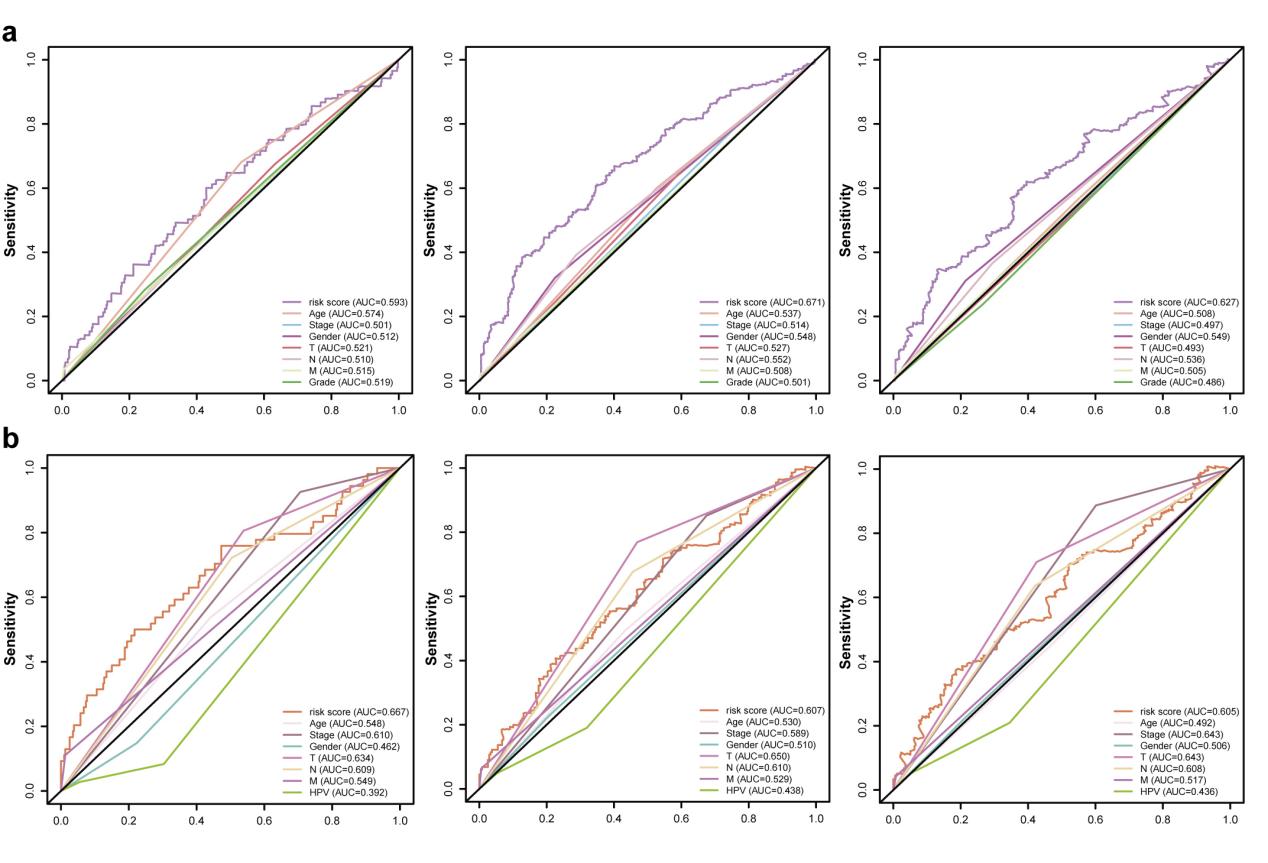


**Fig. S4 Multi-parameter ROC curves of 4-genes model expression.**

The 1, 3 and 5-year multi-parameter ROC curves demonstrated the predictive efficiency of the risk score in (a) TCGA and (b) GEO cohorts.


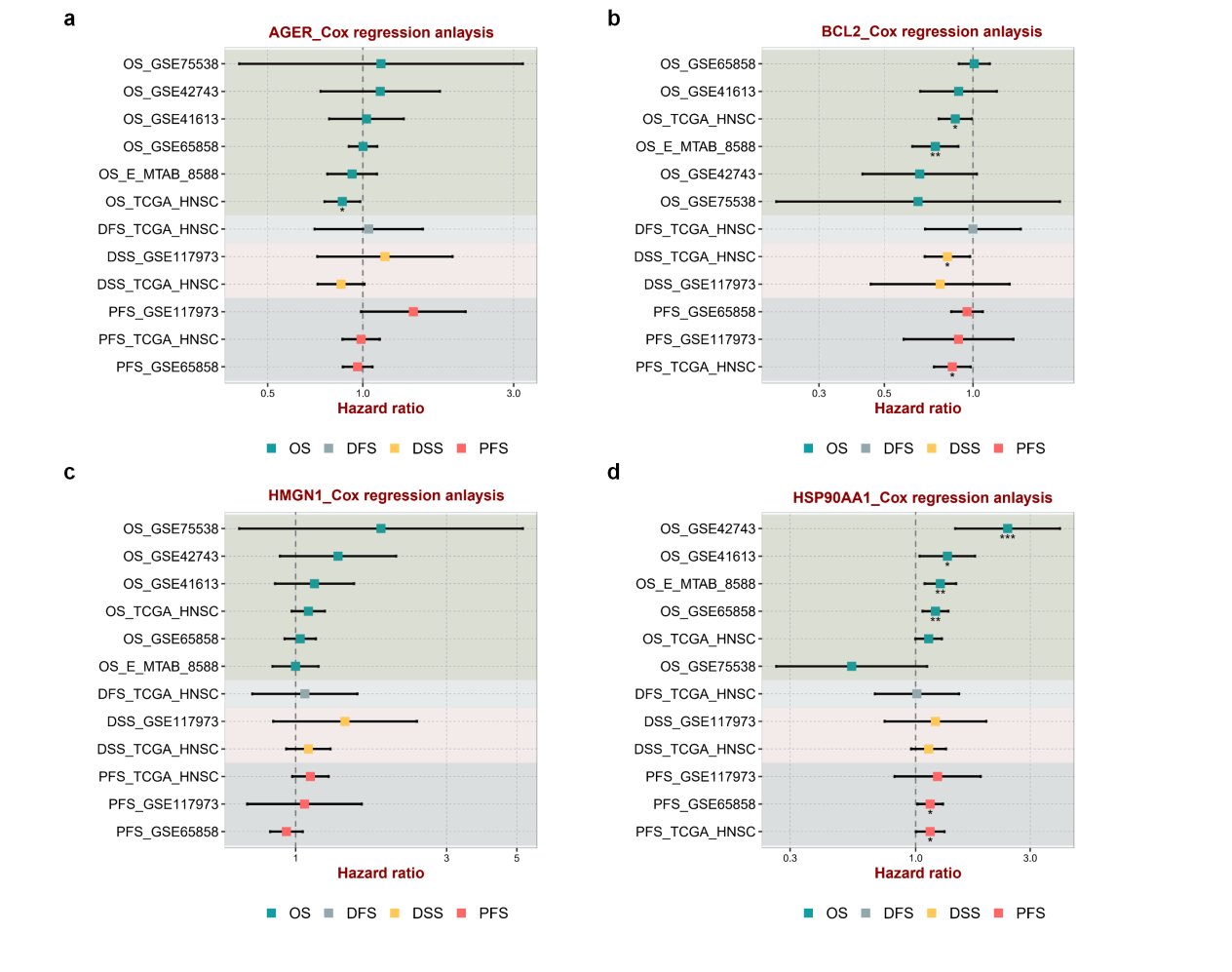


**Fig. S5** **HSP90AA1 may serves as a strong candidate in predicting HNSCC patient outcomes.**

(a-d) Hazard ratio (HR) of overall survival (OS), disease-free survival (DFS), disease-specific survival (DSS) and progression free survival (PFS) of four ICDGs were calculated in the indicated cohorts using Cox regression. Confidence intervals (95%) are presented with HR.


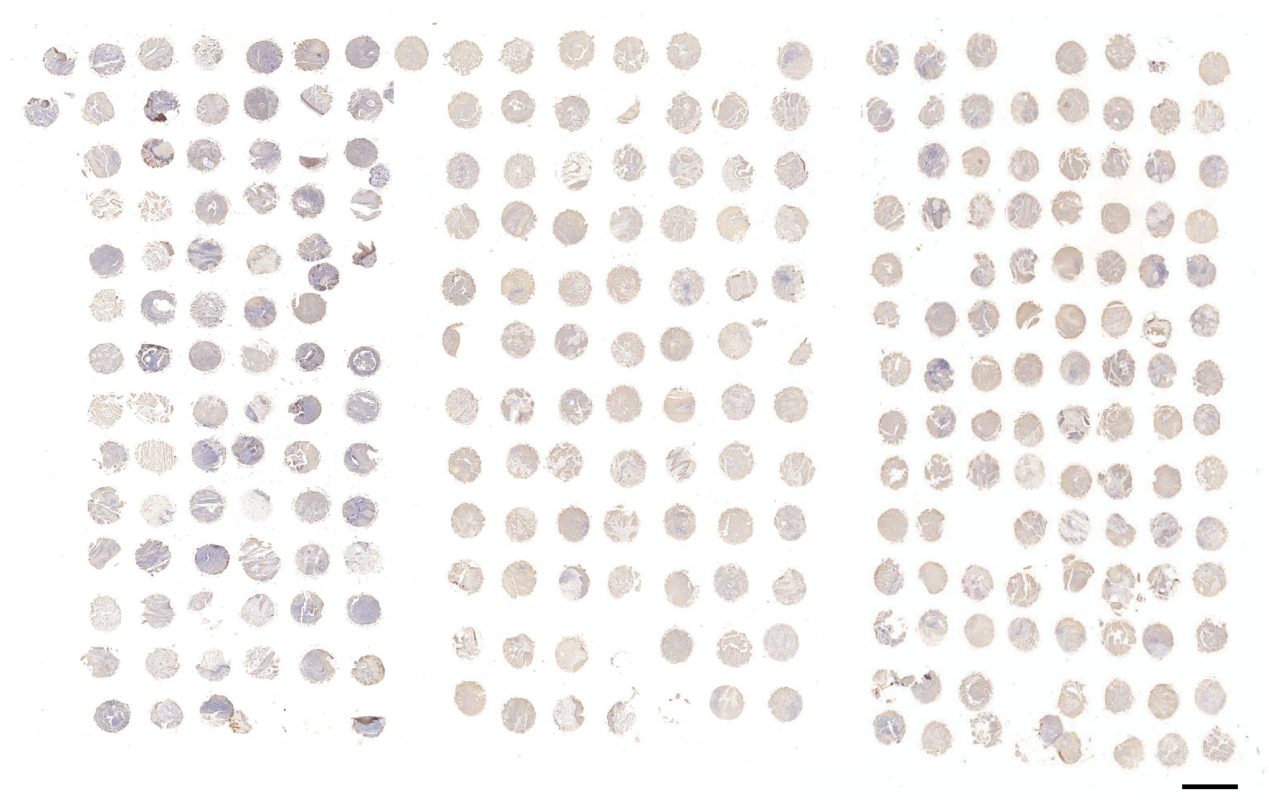


**Fig. S6 IHC assay of HSP90AA1 expression in 208 HNSCC patient tissues.**

A tissue microarray with 208 HNSCC tumor tissues was subjected to IHC assay to determine the expression level of HSP90AA1. Scale bar = 2000 μm.
